# Effects of early pregnancy BMI, mid-gestational weight gain, glucose and lipid levels in pregnancy on offspring’s birth weight and subcutaneous fat: a population-based cohort study

**DOI:** 10.1186/s12884-015-0512-5

**Published:** 2015-04-03

**Authors:** Christine Sommer, Line Sletner, Kjersti Mørkrid, Anne Karen Jenum, Kåre Inge Birkeland

**Affiliations:** Department of Endocrinology, Morbid Obesity and Preventive Medicine, Oslo University Hospital, Postbox 4959, Nydalen, N-0424 Oslo, Norway; Institute of Clinical Medicine, Faculty of Medicine, University of Oslo, Oslo, Norway; Department of Child and Adolescents Medicine, Akershus University Hospital, Lørenskog, Norway; Institute of Health and Society, Department of General Practice, Faculty of Medicine, University of Oslo, Oslo, Norway; Faculty of Health Sciences, Oslo and Akershus University College of Applied Sciences, Oslo, Norway

**Keywords:** Maternal glucose, Maternal lipids, Mid-gestational weight gain, Birth weight, Neonatal adiposity, Subcutaneous fat, Skinfolds, Body composition, Newborn, Multi-ethnic

## Abstract

**Background:**

Maternal glucose and lipid levels are associated with neonatal anthropometry of the offspring, also independently of maternal body mass index (BMI). Gestational weight gain, however, is often not accounted for. The objective was to explore whether the effects of maternal glucose and lipid levels on offspring’s birth weight and subcutaneous fat were independent of early pregnancy BMI and mid-gestational weight gain.

**Methods:**

In a population-based, multi-ethnic, prospective cohort of 699 women and their offspring, maternal anthropometrics were collected in gestational week 15 and 28. Maternal fasting plasma lipids, fasting and 2-hour glucose post 75 g glucose load, were collected in gestational week 28. Maternal risk factors were standardized using z-scores. Outcomes were neonatal birth weight and sum of skinfolds in four different regions.

**Results:**

Mean (standard deviation) birth weight was 3491 ± 498 g and mean sum of skinfolds was 18.2 ± 3.9 mm. Maternal fasting glucose and HDL-cholesterol were predictors of birth weight, and fasting and 2-hour glucose were predictors of neonatal sum of skinfolds, independently of weight gain as well as early pregnancy BMI, gestational week at inclusion, maternal age, parity, smoking status, ethnic origin, gestational age and offspring’s sex. However, weight gain was the strongest independent predictor of both birth weight and neonatal sum of skinfolds, with a 0.21 kg/week increased weight gain giving a 110.7 (95% confidence interval 76.6-144.9) g heavier neonate, and with 0.72 (0.38-1.06) mm larger sum of skinfolds. The effect size of mother’s early pregnancy BMI on birth weight was higher in non-Europeans than in Europeans.

**Conclusions:**

Maternal fasting glucose and HDL-cholesterol were predictors of offspring’s birth weight, and fasting and 2-hour glucose were predictors of neonatal sum of skinfolds, independently of weight gain. Mid-gestational weight gain was a stronger predictor of both birth weight and neonatal sum of skinfolds than early pregnancy BMI, maternal glucose and lipid levels.

## Background

Delivery of macrosomic babies is associated with pregnancy complications such as shoulder dystocia in the offspring [1], cesarean delivery and injuries to the birth canal [2]. Both high and low birth weights have been associated with adverse health outcomes for the child in later life, such as obesity [3] and type 2 diabetes [4]. Although easy to measure, birth weight is generally considered a rough indicator of fetal growth, as the differences in birth weight may be attributed both to differences in fat and lean mass [5]. Fat mass is considered a sensitive marker of the fetal environment and high amounts of fat in the newborn may predispose to obesity and its metabolic complications in later life [6].

In the Hyperglycemia and Adverse Pregnancy Outcome (HAPO) study, a continuous relationship between maternal glucose levels and birth weight was demonstrated, indicating that even moderately elevated glucose levels may increase risk of fetal overgrowth [7]. Pedersen [8] suggested already in 1952 that maternal hyperglycemia transmits to the fetus and induce fetal hyperinsulinemia that stimulates growth and leads to increased birth weight and excessive body fat in the offspring [8]. In concordance with the Pedersen hypothesis, maternal glucose is associated with birth weight [9-11]. Also, studies have found associations between maternal lipids and fetal growth, especially triglycerides and HDL-cholesterol, and one study recently found total cholesterol to be of similar importance as maternal glucose for birth weight [12]. However, high maternal prepregnancy weight and gestational weight gain may result in higher risk of increased birth weight and adverse outcomes than gestational diabetes per se [10,13,14]. Prepregnant BMI is readily accounted for in studies of associations between maternal glucose and offspring’s birth weight. Gestational weight gain, however, is often not accounted for [10], although excessive gestational weight gain has been associated with both gestational diabetes [15,16] and infants born large for gestational age [10,14].

The HAPO study found an association between maternal glucose and neonatal fat mass [7]. However, whether the association between maternal glucose and neonatal fat mass is independent of weight gain in pregnancy, has to our knowledge not been explored. Maternal glucose and lipid levels and their associations with neonatal anthropometrics could therefore be influenced by maternal weight gain in pregnancy.

The objective was to explore whether the effects of maternal glucose and lipid levels on offspring’s birth weight and subcutaneous fat were independent of early pregnancy BMI and mid-gestational weight gain.

## Methods

The details of the STORK Groruddalen study have been described previously [17]. In short, it is a population-based cohort study of healthy pregnant women attending Child Health Clinics for antenatal care in three administrative city districts in Oslo, Norway, May 2008-May 2010. Women were eligible if they: 1) lived in the study districts; 2) planned to give birth at one of two study hospitals; 3) were < 20 weeks pregnant; 4) could communicate in Norwegian or any of the eight translated languages; and 5) were able to give a written consent to participate. To allow for as complete sampling as possible, a minor number of women were included later than 20 weeks: 77 (9.4%) women were included from gestational week 20 to 24, while 11 (1.3%) women were included after gestational week 24. Women with pregestational diabetes or in need of intensive hospital follow-up during pregnancy were excluded. The women were included in gestational week 15 (Visit 1). Measurements were repeated in gestational week 28 (Visit 2), when also an oral glucose tolerance test was performed.

The study was approved by the Norwegian “Regional Committee for Medical and Health Research Ethics South East” and “The Norwegian Data Inspectorate”, and a written consent was obtained for all participants.

### Questionnaire data

Maternal age, parity, smoking status and ethnic origin were collected through interviewer-administered questionnaires at Visit 1. Maternal age was calculated based on date of birth. Parity was dichotomized into nulliparous and parous. Smoking status was collected through two questions: 1) smoker for the last three months prior to pregnancy and 2) smoker during pregnancy. As only 28 women smoked occasionally or daily during pregnancy, we collapsed the two questions before entering it as a dummy variable into the regression analysis. Ethnic origin was defined as country of birth or participant’s mother’s country of birth if the participant’s mother was born outside of Europe or North America, and divided into Europe, South Asia, Middle East, East Asia and South or Central Africa. Three women originating from North America were placed in the Europe category.

### Maternal early pregnancy BMI and mid-gestational weight gain

Height was measured to the nearest 0.1 cm with a fixed stadiometer at Visit 1. Body weight was measured with a calibrated digital scale (Tanita-BC 418 MA, Tanita Corporation, Tokyo, Japan) at Visit 1 and Visit 2. BMI in early pregnancy was calculated from weight and height measured at Visit 1. Pre-pregnancy BMI was calculated from self-reported body weight reported at Visit 1 and height measured at Visit 1. Mid-gestational weight gain was defined as the difference in body weight between Visit 1 and Visit 2, divided by the number of weeks between the two visits for each individual.

### Maternal glucose and lipids

Maternal glucose and lipid levels were measured at Visit 2. Fasting and 2-hour glucose post 75 g glucose load, and fasting total-, HDL- and LDL-cholesterol and triglycerides were measured from venous blood with a colorimetric method (Vitros 5.1 FS, Ortho clinical diagnostics) at the central laboratory. A minority of participants (4.4% for fasting glucose, 7.2% for 2-hour glucose) lacked valid glucose values from the central laboratory. We supplemented missing values with values obtained with a point of care testing device calibrated for plasma (HemoCue 201+, Angelholm, Sweden) (for fasting glucose: n = 20, 2.9%; for 2-hour glucose: n = 38, 5.5%), or if point of care values were missing as well, we used values collected from medical records (for fasting glucose n = 11, 1.6%; for 2-hour glucose n = 12, 1.7%). Women diagnosed with gestational diabetes by the World Health Organization (WHO) 1999 criteria (fasting plasma glucose ≥ 7.0 mmol/L or 2-hour glucose ≥ 7.8 mmol/L) at Visit 2 received lifestyle advice and were referred to their General Practitioner for follow-up if 2-hour glucose was <9.0 mmol/L or to hospital care if 2-hour glucose was ≥ 9.0 mmol/L [18].

### Neonatal variables

To be able to compare our results with similar studies, gestational week was calculated from the first day of the woman’s last menstrual period (LMP) and term was calculated as date of LMP +282 days (standard in Norway). Ultrasound term (from routine scan) was used for 24 (3.4%) women where the LMP date was missing or differed ≥ 14 days from ultrasound term [19]. The outcome birth weight was measured with calibrated electronic scales immediately after birth [17]. To assess neonatal subcutaneous fat, we measured skinfolds to the nearest 0.2 mm, with a skinfold caliper (Holtain T/W Skinfold Caliper, Holtain Ltd., Crymych, UK) at subscapular, suprailiac, thigh and triceps sites within 72 hours after birth. We measured all skinfolds twice and used the average. The outcome sum of skinfolds, was calculated by summarizing the four skinfold sites. Inter-rater variability (measured as % Technical Error of Measurement) for the skinfold measurements ranged from 8-13%, while intra-rater variability was less than 5% in all measurements [19].

### Statistical analysis

**Figure 1:**
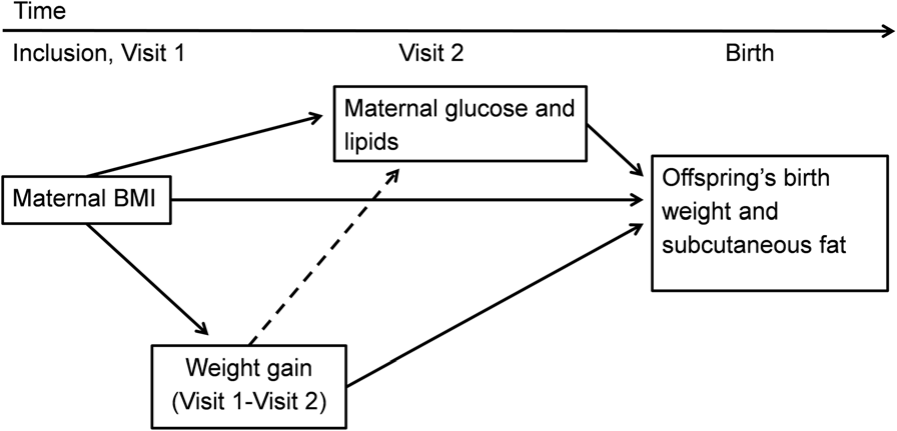
**Hypothesized timeline of the multiple regression analysis.** Solid lines indicate already established relationships and dotted lines the hypothesized relationships. We hypothesized that maternal early pregnancy BMI and mid-gestational weight gain could modify the effects of maternal glucose and lipids on offspring’s birth weight and neonatal subcutaneous fat.

We used maternal early pregnancy BMI from Visit 1, maternal weight gain from Visit 1 to Visit 2, and maternal glucose and lipid level measured at Visit 2 to meet with assumptions of temporality (Figure 1). All maternal risk factor variables were standardized using z-score to ease comparison of their effects on the outcomes. We performed simple univariate linear regression analyses (Model 0) to explore associations between maternal risk factor variables and the outcomes birth weight and sum of skinfolds. We performed multiple linear regressions separately for the outcomes birth weight and sum of skinfolds to explore independent effects of maternal risk factor variables. In Model 1, maternal glucose and lipid variables that correlated with the respective outcomes with a P-value < 0.2 in Model 0, were entered simultaneously into a multiple regression and adjusted for gestational week at inclusion, maternal age, parity, smoking status, ethnic origin gestational age and offspring’s sex. To see if BMI or weight gain influenced the effect of maternal glucose and lipid levels on the outcomes, we additionally adjusted for early pregnancy BMI in Model 2, and additionally for mid-gestational weight gain in Model 3. We explored possible interactions between the maternal risk factor variables and ethnic origin, and between the maternal risk factor variables and offspring’s sex, by including interaction terms into the multiple regressions for both outcomes. We performed sensitivity analyses by repeating the multiple regression analysis after excluding 83 women who were diagnosed with gestational diabetes; by using pre-pregnancy (self-reported) weight gain to gestational week 28 and; by analyzing normal weight and overweight women separately, according to classifications by the WHO. All statistical analyses were performed using IBM SPSS Statistics 21. We used the lincom command in StataIC 12 to calculate predicted birth weight and sum of skinfolds for the sole and combined effects of significant risk factor variables, based on Model 3 of the multiple regression analysis separately for each outcome. To estimate an “optimal” birth weight and sum of skinfolds in the offspring, we defined an optimal early pregnancy BMI as 23 kg/m^2^ and an optimal weight gain was defined as 0.42 kg/week in accordance with recommendations from the Institute of Medicine [20]. To estimate high or low maternal glucose and lipid levels we used cut offs at the 90^th^ or the 10^th^ percentile.

### Flow of the cohort

The participation rate was 74%, varying from 63.9 to 82.6 across ethnic groups [17]. Age did not differ between the 823 who participated and the 291 who chose not to participate. South Asians who did not participate were more parous than those who participated, while there was no difference within the remaining ethnic groups [17]. The study cohort was representative for the main ethnic groups, and there were no ethnic differences in reasons for exclusion [17,21].

**Figure 2:**
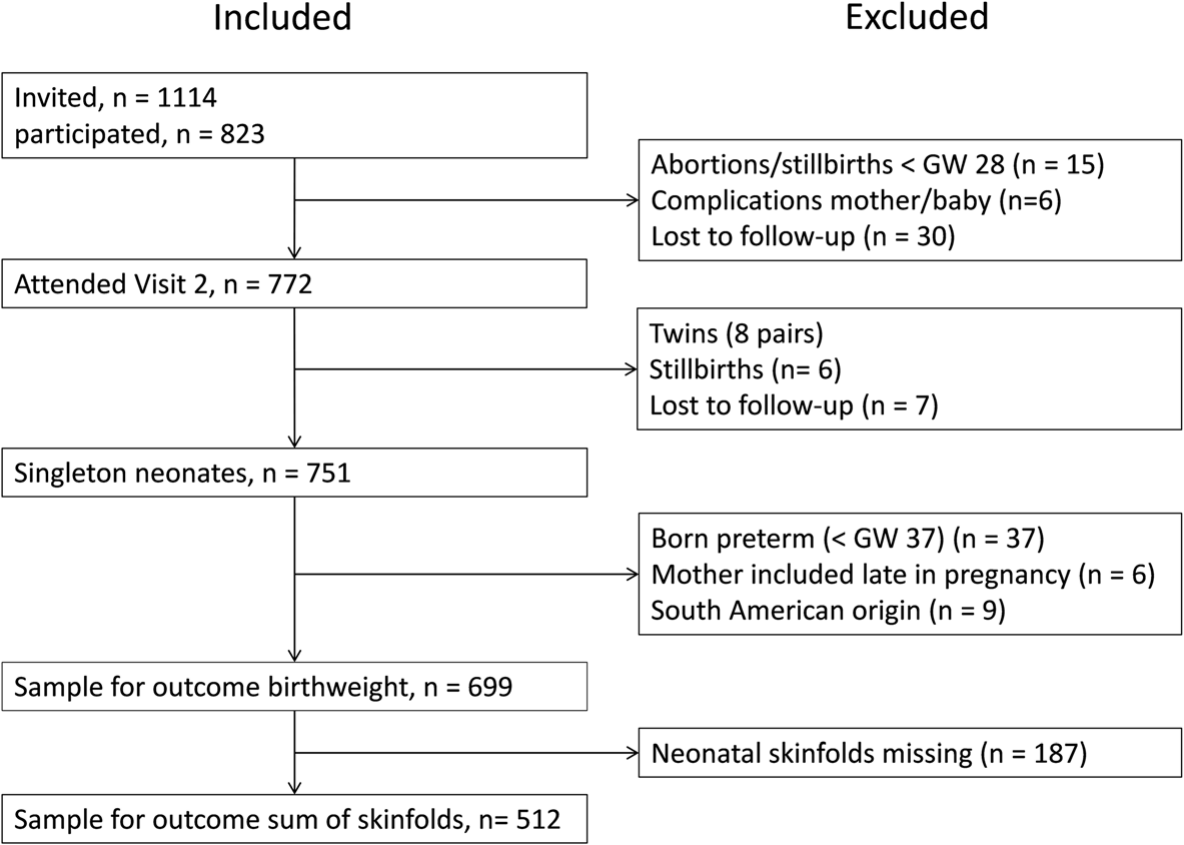
**Flow of the cohort.**

Of the 823 women originally included in the study, 751 mothers of singleton neonates met at Visit 2 (Figure 2). We excluded 37 women with preterm births (gestational week < 37), six who were included after gestational week 24 and nine with a South American origin due to heterogeneity, leaving us with a sample of 699 women and their offspring. We found no difference between the 124 excluded women and the 699 included women in age, parity, education level or duration of residence in Norway for immigrants. A higher proportion of the excluded women were single (8.9% vs 3.0%, p = 0.037) and originated from South or Central Africa (14.5% vs 6.3%, p = <0.001). Neonatal skinfold measurements were missing for 187 offspring mainly due to study staff not being notified of the birth within 72 hours [19]. With sum of skinfolds as the outcome, our sample therefore comprised 512 women and their offspring (Figure 2). We did not find any differences in the characteristics listed in Table 1 between mother-offspring pairs with and without neonatal skinfold measurements.

**Table 1:** **Characteristics of the sample**

	**Sample (n = 699)**
**Maternal characteristics**	
Age (years)	29.3 ± 4.8
Nulliparous	319 (45.6)
Ethnic origin	
Europe	335 (47.9)
South Asia	173 (24.7)
Middle East	112 (16.0)
East Asia	35 (5.0)
South or Central Africa	44 (6.3)
Prepregnancy BMI (kg/m^2^)	24.6 ± 4.8
Early pregnancy BMI, Visit 1 (kg/m ^2^)	25.3 ± 4.8
Weight gain, Visit 1-2 (kg/week)	0.51 ± 0.21
Visit 1 (gestational week)	15 ± 3
Visit 2 (gestational week)	29 ± 1
Smoked 3 months prior to pregnancy	123 (17.6)
Smoked during pregnancy	28 (4.0)
**Neonatal characteristics**	
Gestational age at birth (days)	281 ± 9
Female sex	337 (49.7)
Birth weight (g)	3491 ± 498
Neonatal sum of skinfolds (mm) ^a^	18.2 ± 3.9
Mean skinfold triceps (mm) ^a^	4.4 ± 1.0
Mean skinfold thigh (mm) ^a^	5.9 ± 1.4
Mean skinfold suprailiac crest (mm) ^a^	3.5 ± 0.9
Mean skinfold subscapular (mm) ^a^	4.4 ± 1.1
**Maternal glucose and lipids**	
*Glucose*	
*Fasting glucose (mmol/L)*	
Visit 1	4.4 ± 0.4
Visit 2	4.4 ± 0.5
2-hour glucose (mmol/L)	
Visit 2	5.8 ± 1.5
Gestational diabetes	84 (12.2)
*Lipids*	
Total cholesterol (mmol/L)	
Visit 1	5.0 ± 0.9
Visit 2	6.2 ± 1.1
HDL-cholesterol (mmol/L)	
Visit 1	1.73 ± 0.39
Visit 2	1.93 ± 0.45
LDL-cholesterol (mmol/L)	
Visit 1	2.71 ± 0.73
Visit 2	3.44 ± 0.99
Triglycerides (mmol/L)	
Visit 1	1.31 ± 0.55
Visit 2	1.98 ± 0.69

Data are mean ± standard deviation or n (%).

^a^ n = 512.

## Results

The mean maternal age was 29.3 ± 4.9 years, 45.6% (n = 319) were nulliparous and 47.9% (n = 335) had a European ethnic origin (Table 1). Mean self-reported prepregnancy BMI was 24.6 ± 4.8 kg/m^2^, early pregnancy BMI at Visit 1 was 25.3 ± 4.8 and mean mid-gestational weight gain between Visit 1 and Visit 2 was 0.51 ± 0.21 kg/week (Table 1). The women were included in gestational week 15 ± 3, while maternal glucose and lipids were measured in gestational week 29 ± 1. The mean gestational age of the neonates was 281 ± 9 days, birth weight was 3491 ± 498 g and mean sum of skinfolds was 18.2 ± 3.9 mm (Table 1). Girls had lower birth weight (3420 ± 491 vs. 3559 ± 491 g) and had a larger sum of skinfold than boys (18.6 ± 4.0 mm vs. 17.8 ± 3.8 mm).

### Predictors of offspring’s birth weight and sum of skinfolds

In univariate simple regression analyses (Table 2, Model 1), maternal fasting glucose, early pregnancy BMI, and mid-gestational weight gain were all associated to offspring’s birth weight (all P < 0.001), while the associations with HDL-cholesterol (P = 0.023), 2-h glucose (P = 0.069) and triglyceride level (P = 0.105) were non-significant. With sum of skinfolds as the outcome, both fasting and 2-h glucose (both P < 0.001), early pregnancy BMI (P < 0.001) and mid-gestational weight gain (P = 0.006) were associated, while the effect sizes of triglycerides (P = 0.025) and HDL-cholesterol (P = 0.199) on sum of skinfolds were weaker.

**Table 2:** **Univariate simple and multiple linear regressions of maternal risk factor variables (z-score) on offspring’s birth weight (g) and sum of skinfolds (mm)**

	**Model 0**		**Model 1**		**Model 2**		**Model 3**		**Model 4**	
	**Simple**		**Adjusted**		**Model 1 + BMI**		**Model 2 + Weight gain**		**Model 3 + interaction term**	
	**β**	**P**	**β**	**(95% CI)**	**β**	**(95% CI)**	**β**	**(95% CI)**	**β**	**(95% CI)**
**Birth weight (g)**										
Fasting glucose	**83.5**	<0.001	**79,5**	(42.5 to 115.1)	**64,9**	(26.5 to 103.3)	**43,2**	(5.4 to 81.1)	**43,7**	(5.9 to 81.5)
2-hour glucose	34.7	0.069	11,4	(-24.7 to 47.5)	9,7	(-26.2 to 45.7)	15,9	(-19.1 to 50.8)	16,6	(-18.3 to 51.5)
HDL-cholesterol	**−43.3**	0.023	−28,1	(-63.1 to 6.9)	−27,2	(-62.0 to 7.6)	**−41,6**	(-75.6 to -7.5)	**−44,5**	(-78.6 to -10.4)
Triglycerides	30,8	0.105	32,5	(-4.2 to 69.2)	27,8	(-8.7 to 64.5)	35,0	(-0.7 to 70.6)	34,9	(-0.6 to 70.5)
BMI in early pregnancy	**127.2**	<0.001			**49,5**	(12.6 to 86.4)	**68,3**	(32.0 to 104.5)		
BMI in Europeans									**33,5**	(17.1 to 50.0)
BMI in non-Europeans									**103,7**	(54.7 to 152.7)
Weight gain ^a^	**79.5**	<0.001					**110,7**	(76.6 to 144.9)	**111,3**	(77.2 to 145.3)
**Sum of skinfolds (mm)**										
Fasting glucose	**0.92**	<0.001	**0,75**	(0.39 to 1.12	**0,73**	(0.36 to 1.11)	**0,57**	(0.19 to 0.95)	**0,58**	(0.20 to 0.96)
2-hour glucose	**0.73**	<0.001	**0,40**	(0.05 to 0.75)	**0,40**	(0.05 to 0.75)	**0,44**	(0.09 to 0.78)	**0,44**	(0.09 to 0.78)
HDL-cholesterol	−0,22	0.199	−0,09	(-0.43 to 0.25)	−0,09	(-0.43 to 0.26)	−0,18	(-0.52 to 0.16)	−0,19	(-0.54 to 0.15)
Triglycerides	**0.39**	0.025	0,21	(-0.15 to 0.57)	0,20	(-0.15 to 0.56)	0,24	(-0.11 to 0.60)	0,25	(-0.11 to 0.60)
BMI in early pregnancy	**0.77**	<0.001			0,08	(-0.30 to 0.47)	0,25	(-0.13 to 0.64)		
BMI in Europeans									0,10	(-0.08 to 0.28)
BMI in non-Europeans									0,41	(-0.11 to 0.92)
Weight gain ^a^	**0.48**	0.006					**0,72**	(0.38 to 1.06)		(0.38 to 1.06)

Maternal risk factor variables are expressed as standard deviations (SDs). Values are β and P-value in Model 0, and in the remaining models; β (95% CI), with 1SD increase in maternal risk factor variables representing a unit change in birth weight (g) or sum of skinfolds (mm).

Model 0 are simple regression analyses, listed variables analyzed separately.

Model 1 is a multiple regression of the risk factor variables entered simultaneously, adjusted for gestational week at inclusion, maternal age, parity, smoking status, ethnic origin, offspring’s sex and gestational age.

Model 2 = model 1+ early pregnancy BMI.

Model 3 = model 2 + weight gain.

Model 4 = model 3 + interaction term, BMI X European ethnic origin. β’s for BMI are presented separately for Europeans and non-Europeans in Model 4.

Bold β value indicates P < 0.05.

^a^ Weight gain from Visit 1 (gestational week 15) to Visit 2 (gestational week 28).

In the multiple regression analyses adjusted for relevant covariates (Table 2, Model 1) fasting glucose was a significant predictor of birth weight and both fasting and 2-h glucose were significant predictors of sum of skinfolds. Early pregnancy BMI was a significant and independent predictor of offspring’s birth weight, but not for sum of skinfolds (Table 2, Model 2). After adjusting for early pregnancy BMI (Table 2, Model 2) the effect of fasting glucose on birth weight decreased, while the effects of fasting and 2-hour glucose on sum of skinfolds were unchanged. Mid-gestational weight gain was a significant and independent predictor of both offspring’s birth weight and sum of skinfolds (Table 2, Model 3). After adjusting for weight gain, the effect of fasting glucose on both birth weight and sum of skinfolds decreased, but remained an independent predictor of both outcomes, while the effect of 2-hour glucose on sum of skinfolds was slightly increased and thereby remained an independent predictor (Table 2). HDL-cholesterol was not an independent predictor of birth weight until weight gain was adjusted for, while 2-hour glucose and triglycerides were not independently associated with birth weight (Table 2). None of the lipid parameters were independent predictors of sum of skinfolds (Table 2).

As women who were diagnosed with gestational diabetes received lifestyle advice at time of diagnose, we repeated the analysis without these 83 women. The effects of the risk factor variables were unchanged except for the effect of triglycerides on neonatal sum of skinfolds, where the effect size increased and the association became significant (β =0.45 (95% confidence interval 0.04-0.85). Using weight gain from pre-pregnancy (self-reported) to gestational week 28 did not change the effect estimates of the independent predictors for neither outcome (data not shown). Analyzing normal weight and overweight women separately, using model 3 of the regression, did not change the effect estimates of the independent predictors for neither outcome (data not shown).

### Impact of ethnic origin

Compared to offspring of European mothers, the mean birth weight was 325 (408-243) g lower in offspring of South Asian mothers, 168 (263-73) g lower in offspring of Middle Eastern mothers, 239 (389-89) g lower in offspring of East Asian mothers and 161 (303-19) g lower in offspring of African mothers. The mean sum of skinfolds was significantly lower by 1.7 (2.5-0.9) mm in offspring of South Asian mothers and by 1.2 (2.2-0.2) mm in offspring of Middle Eastern mothers than in offspring of European mothers.

We found no interactions between ethnic origin and the risk factor variables with sum of skinfolds as the outcome. With birth weight as the outcome, there was a significant interaction between ethnic origin and maternal early pregnancy BMI (Table 2, Model 4). Among Europeans, a 4.8 kg/m^2^ higher BMI resulted in a 33.5 (17.1-50.0) g heavier offspring, while for non-Europeans it resulted in a 103.7 (54.7-152.7) g heavier offspring (Table 2, Model 4). The effect size of BMI on birth weight was higher in all ethnic minority sub-groups (data not shown). Adding the interaction term to the final model of the multiple regression analysis did not substantially change the effect estimates for the other risk factor variables (Table 2, Model 4).

### Combined effects

**Figure 3:**
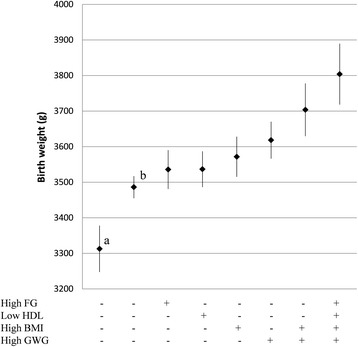
**Predicted birth weight for the sole and combined effects of risk factor variables.** Based on estimations by the adjusted multiple regression Model 3. High FG (5.0 mmol/L), BMI (31.6 kg/m2) and GWG (0.78 kg/week) were defined as their respective 90 percentile value, low HDL (1.4 mmol/L) as its 10 percentile value. + indicates presence and - absence of the predictor, remaining variables in the model were set at sample mean. Diamonds are predicted mean birth weight and error bars are 95% CI’s. FG = fasting glucose, HDL = HDL - cholesterol, GWG = gestational weight gain. ^a^ Predicted birth weight if maternal BMI was 23 kg/m2, GWG was 0.42 kg/week (according to recommendations from the Institute of Medicine) [20], FG = 3.9 mmol/L (10 percentile) and HDL = 2.5 mmol/L (90 percentile). ^b^ Predicted birth weight if all variables in the multiple regression model were set at sample mean.

**Figure 4:**
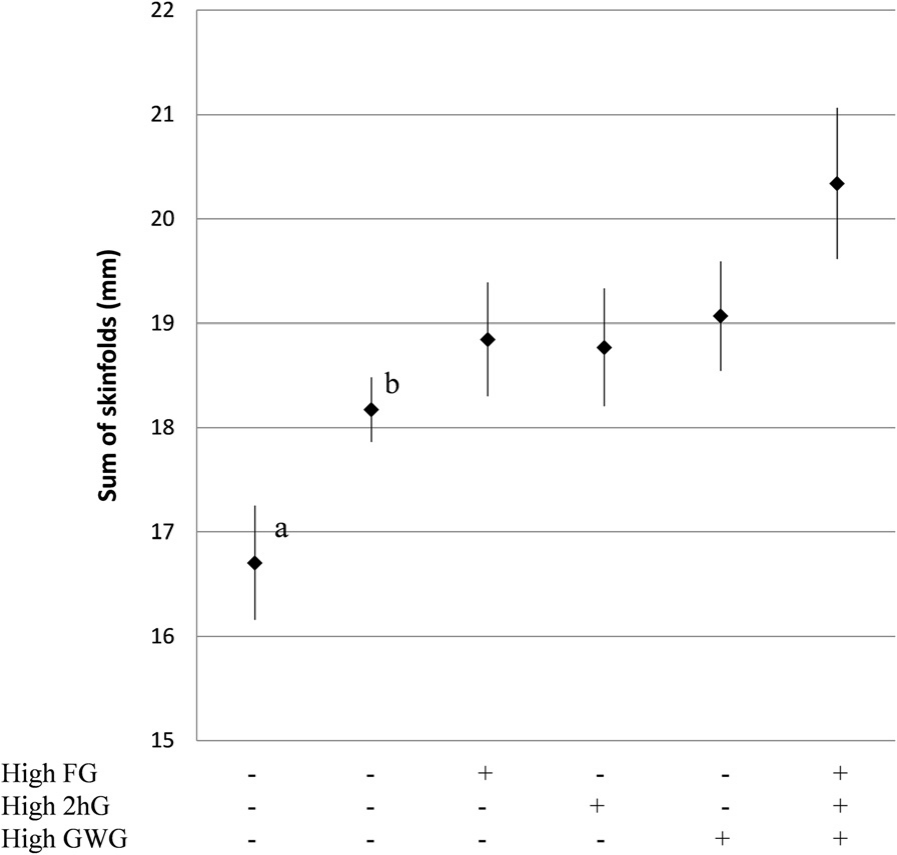
**Predicted sum of skinfolds for the sole and combined effects of risk factor variables.** Based on estimations by the adjusted multiple regression Model 3. High FG (5.0 mmol/L), 2hG (7.9 mmol/L) and GWG (0.77 kg/week) were defined as their respective 90 percentile value. + indicates presence and - absence of the predictor, remaining variables in the model were set at sample mean. Diamonds are predicted mean sum of skinfolds and error bars are 95% CI’s. FG = fasting glucose, 2hG = 2 - hour glucose, GWG = gestational weight gain. ^a^ Predicted sum of skinfolds if maternal BMI was 23 kg/m2, GWG was 0.42 kg/week (according to recommendations from the Institute of Medicine) [20], FG = 3.9 mmol/L (10 percentile) and 2hG = 4.1 mmol/L (10 percentile). ^b^Predicted sum of skinfolds if all variables in the multiple regression model were set at sample mean.

Mid-gestational weight gain was the strongest independent predictor of both birth weight and sum of skinfolds (Table 2), while the other maternal predictors had more similar effects on the outcomes. As maternal fasting glucose, HDL-cholesterol, BMI and weight gain were all independent predictors of birth weight, high levels of fasting glucose, BMI and weight gain and low levels of HDL-cholesterol combined (Figure 3, diamond to the right) gave a heavier neonate than if all factors were absent (Figure 3, diamond to the left). Likewise, since fasting glucose, 2-hour glucose and weight gain were all independent predictors of neonatal sum of skinfolds, estimations based on a combination of high levels of these maternal factors (Figure 4, diamond to the right) gave a higher amount of subcutaneous fat in the neonate than if all factors were absent (Figure 4, diamond to the left).

## Discussion

In this multiethnic, population-based cohort of pregnant women, we found maternal fasting glucose and HDL-cholesterol in gestational week 28 to be important predictors of birth weight, independently of the mother’s early pregnancy BMI and mid-gestational weight gain. Maternal fasting and 2-hour glucose in gestational week 28 were predictors of neonatal sum of skinfolds, independently of BMI and mid-gestational weight gain. However, mid-gestational weight gain was a stronger independent predictor than maternal glucose, lipids and early pregnancy BMI, for both birth weight and sum of skinfolds in the offspring. Mid-gestational weight gain and fasting glucose were the only risk factor variables that were independent predictors for both outcomes. Furthermore, the effect size of mother’s early pregnancy BMI on birth weight was higher in non-Europeans than in Europeans.

Despite a strong focus on maternal glycemia after the HAPO study and the proposed new criteria for gestational diabetes [22], few studies have explored the effects of glucose and lipid levels on the newborn’s anthropometrics independently of gestational weight gain. Retnakaran and coworkers [14] also found that weight gain and BMI were the most important determinants of birth weight and large for gestational age offspring, independently of maternal glucose intolerance and lipid levels.

Consistent with our findings, several studies have found a relationship between maternal fasting glucose and offspring’s birth weight [12,23,24], and both maternal fasting glucose [7] and 2-hour glucose [25] have been associated with neonatal adiposity. In our study, 2-hour glucose was independently associated with offspring’s subcutaneous fat, but not birth weight.

As found in other studies [26-28], HDL-cholesterol was inversely related to birth weight in our study. One study [27] found an inverse association between HDL-cholesterol and birth weight only in overweight and obese women and suggested that the effect of HDL-cholesterol was modified by BMI. However, we found the same effect of HDL on birth weight in normal weight women as in overweight women. A study of underweight and nutrient deficient women [12] did not find an association between HDL-cholesterol and birth weight, but this study did not adjust for weight gain and had a very different study population.

One of the few studies who adjusted for weight gain in pregnancy [14] did not find any effect of HDL-cholesterol, LDL-cholesterol or triglycerides on birth weight, while others have found a positive association between triglycerides and birth weight [29,30]. It is possible that the effect of triglycerides was neutralized by the glucose variables in our multiple regression, as triglyceride levels will increase when high insulin levels are present (e.g. in an insulin resistant state). However, the unadjusted effect of triglycerides on birth weight was relatively weak in our study.

We found no independent effect of BMI on neonatal sum of skinfolds, while other studies have found an association between maternal BMI and neonatal adiposity [9,31]. In our sample, the association between BMI and neonatal sum of skinfolds disappeared when fasting glucose was adjusted for, also when we excluded women who were diagnosed with gestational diabetes. BMI reflects maternal body size, such as muscle mass, skeletal size and body-build in general, and not merely maternal adiposity. Maternal body size may affect the size of the offspring due to genetic traits, but also, a small maternal body size may constrain the size of the offspring [32]. Our finding that BMI had a different effect on birth weight in non-Europeans than in Europeans, is supported by other studies who also found interactions between ethnicity and BMI in relation to prevalence of diabetes [18], gestational diabetes [33] as well as the risk of offspring born large for gestational age in women with gestational diabetes [34]. The fact that this interaction between BMI and ethnic origin is found in relation to several outcomes could mean that non-Europeans have a lower tolerance of adiposity [18], or that BMI is a poor measure of adiposity across ethnic groups, or possibly a combination of both. This is especially concerning considering the higher prevalence of gestational diabetes and pre-pregnant BMI found in non-European ethnic groups in the STORK Groruddalen study [15,21].

Major strengths of the study are the high participation rate and the inclusion of ethnic minority groups. Our study sample is considered representative for the major ethnic groups of pregnant women living in Norway [17]. To our knowledge, this is the first study to show that the association between maternal glucose and neonatal skinfolds is independent not only of BMI, but also of weight gain in pregnancy.

A limitation to our study is that the observational design cannot prove a causal effect between the maternal factors and offspring’s anthropometry. Another limitation is the lack of control with the different components of the weight gain. However, weight gain is easy to measure, reflects fat gain, and is not likely to cause bias. Further, the relative weight of the fetus will not be as pronounced in gestational week 28 as compared to later in pregnancy. Using weight gain up to measurement of maternal glucose and lipid levels allowed us to comply with assumptions of temporality. BMI is generally thought to reflect adiposity and disease risk differently in Asians [35]. However, we found the same interaction in all ethnic minority sub groups, indicating that the interaction we found was not a result of the problem with using BMI in Asians. In addition, the interaction between BMI and ethnic origin found in our sample did not change the effect of fasting glucose, HDL-cholesterol or weight gain on birth weight.

## Conclusions

Our results suggest that mid-gestational weight gain could be more important than hyperglycemia in gestational week 28 in relation to offspring’s birth weight and subcutaneous fat. However, maternal prepregnancy BMI, weight gain, glucose and lipid levels are all factors that might benefit from lifestyle advice directed at a healthy diet and increased physical activity, and hence these should, ideally, be optimized before conception. However, since health care workers often meet women when they are already pregnant, promoting an adequate weight gain during pregnancy may be one of the most important modifiable factors of birth weight and subcutaneous fat of the newborn. Future research should explore the long term effects of maternal glucose and lipids in pregnancy, prepregnant obesity and gestational weight gain on the offspring’s health in childhood and adult life.

## Abbreviations

BMI: Body mass index
HAPO: Hyperglycemia and Adverse Pregnancy Outcome
LMP: Last menstrual period
